# An American Text-book of Physiology

**Published:** 1901-08

**Authors:** 


An American Text-book of Physiology.—By H. P. Bowditch, M. D.; John G. Curtis, M. D.; H. H. Donaldson, M. D.; W. H. Howell, M. D.; Fred S. Lee, Ph. D.; W. P. Lombard, M. D.; G. Lusk, Ph. D., F. R. S.; W. T. Porter, M. D.; E. T. Reichert, M. D.; Henry Sewall, M. D. Edited by William H. Howell, Ph. D., M. D., Professor of Physiology in the Johns-Hopkins University, Baltimore, M. D. Second edition; revised; Vol. I., blood, lymph and circulation; Secretion, digestion and nutrition; respiration and animal heat; chemistry of the body. Pages, 598. Price, $3.00. Philadelphia: W. B. Saunders & Co. 1900.

The change from a one-volume to a two-volume work has proved convenient to students and not objectionable to the profession. The text in this edition has been thoroughly revised and brought up to date. The section on the central nervous system in Volume II, and the short section upon the modern ideas and nomenclature of physical chemistry, with reference especially to the process of osmosis and diffusion in Volume I, contain much that is new. The former is by Donaldson and the latter by Howell, of the Johns-Hopkins. The section on "Animal Heat" is by Edward T. Reichert, of the University of Pennsylvania. Graham Lusk contributed the section on "The Chemistry of the Animal Body."

There is an ample index to the volume, and the illustrations are of the most modern and useful kind. Volume II is now ready.

